# Soursop-Derived Gut Metabolites Restore Adipose Metabolic Homeostasis and Attenuate Fructose-Induced Adipotoxicity: Mechanistic Insights into the Soursop–Gut–Adipose Axis

**DOI:** 10.3390/antiox15091106

**Published:** 2026-09-02

**Authors:** Ochuko L. Erukainure, Chika I. Chukwuma

**Affiliations:** 1 Department of Microbiology, School of Life Sciences, University of KwaZulu-Natal, Durban 4000, South Africa; 2 Department of Biochemistry, School of Life Sciences, University of KwaZulu-Natal, Durban 4000, South Africa; 3 Centre for Quality of Health and Living (CQHL), Faculty of Health and Environmental Sciences, Central University of Technology, Bloemfontein 9301, South Africa; cchukwuma@cut.ac.za

**Keywords:** adipotoxicity, glucose metabolism, gut–adipose axis, gut microbiota, lipid metabolism, soursop

## Abstract

Adipotoxicity is a major contributor to insulin resistance and type 2 diabetes, and increasing evidence highlights the gut–adipose axis as a promising therapeutic target. Soursop (*Annona muricata*) is rich in phytochemicals that can be biotransformed by the gut microbiota into bioactive metabolites with metabolic benefits. The present study investigated whether metabolites generated by in vitro fecal fermentation of soursop fruit (SWSF) and peel (SWSFP) protect against fructose-induced adipotoxicity. SWSF and SWSFP were fermented with rat fecal microbiota, and the resulting metabolites were evaluated in an ex vivo fructose-induced adipotoxicity model using perigonadal white adipose tissue. Activities of enzymes involved in glucose metabolism, the polyol pathway, glutathione metabolism, glyoxalase-1 activity, purinergic signaling, and inflammatory lipid metabolism were determined. GC–MS-based metabolomics and pathway enrichment analyses were performed on fecal and adipose tissues. Soursop fermentation significantly remodeled the fecal metabolome, enriching metabolites associated with fatty acid metabolism, glycerolipid metabolism, β-oxidation, sterol metabolism, and arachidonic acid metabolism. Fructose-induced adipotoxicity disrupted glucose metabolism, activated the polyol pathway, impaired glutathione metabolism and glyoxalase-1 activity, suppressed ATPase and ENTPDase activities, and elevated 5-LOX and 12/15-LOX activities. Treatment with soursop-enriched fecal metabolites significantly reversed these alterations in a dose-dependent manner. Adipose metabolomics further demonstrated restoration of pathways associated with fatty acid biosynthesis, mitochondrial β-oxidation, glycerolipid metabolism, steroid biosynthesis, and polyunsaturated fatty acid metabolism, indicating improved lipid homeostasis and reduced inflammatory lipid signaling. SWSF and SWSFP generally exhibited greater metabolic protection than the reference antioxidant compound, gallic acid. Soursop-derived gut metabolites attenuate fructose-induced adipotoxicity by coordinately restoring glucose metabolism, redox homeostasis, carbonyl detoxification, purinergic signaling, and lipid metabolism through the gut–adipose axis. These results suggest soursop as a potential functional food for preventing and managing adipose tissue dysfunction and metabolic disorders.

## 1. Introduction

Adipotoxicity is increasingly recognized as a central mechanism in the pathogenesis and progression of adipose tissue dysfunction, leading to systemic metabolic dysregulation and associated metabolic complications [1,2]. It is characterized by impaired lipid storage and mobilization, insulin resistance, oxidative stress, chronic low-grade inflammation, and altered adipokine secretion within the adipose tissue. This dysfunction may promote lipid overflow and the ectopic accumulation of toxic lipid species in non-adipose organs, including the liver, skeletal muscle, and pancreas, resulting in lipotoxicity and impaired cellular function [3]. These toxic lipid species have been implicated in the pathogenesis of insulin resistance and as contributing to the development of type 2 diabetes (T2D) [3,4,5]. Adipotoxicity also triggers chronic low-grade inflammation by enhancing the secretion of pro-inflammatory cytokines, including tumor necrosis factor-α (TNF-α) and interleukin-6 (IL-6) [6]. These pathological events collectively drive the progression of metabolic syndrome, characterized by dyslipidemia, hypertension, impaired glucose homeostasis, and an increased risk of cardiovascular disease.

The gut–adipose axis represents a dynamic bidirectional network of biochemical, metabolic, immune, and endocrine interactions between the gastrointestinal tract and adipose tissue that plays a central role in maintaining systemic metabolic homeostasis [7]. Increasing evidence indicates that modulation of this axis can attenuate adipotoxicity by preserving gut barrier integrity, regulating microbial composition, and promoting the production of bioactive microbial metabolites, particularly short-chain fatty acids (SCFAs) [8,9]. SCFAs, including acetate, propionate, and butyrate, activate G-protein-coupled receptors such as GPR41 and GPR43 on adipocytes, thereby enhancing insulin sensitivity, promoting lipid oxidation, improving adipocyte differentiation, and suppressing excessive lipolysis that contributes to free fatty acid overflow and ectopic lipid deposition [10,11,12]. Furthermore, maintenance of intestinal barrier function limits the translocation of lipopolysaccharides (LPS) into the circulation, thereby preventing metabolic endotoxemia, chronic low-grade inflammation, and macrophage infiltration of adipose tissue [13,14]. The gut microbiota also regulates bile acid metabolism, which influences brown adipose tissue activation and thermogenesis through bile acid receptor signaling, increasing energy expenditure and reducing lipid accumulation in peripheral tissues [15]. In addition, gut-derived metabolites and postbiotic factors modulate adipose tissue immune responses by promoting polarization of macrophages toward an anti-inflammatory M2 phenotype while enhancing regulatory T-cell activity, thereby suppressing the chronic inflammatory milieu associated with adipose dysfunction [16]. Synergistically, these interconnected mechanisms indicate the gut–adipose axis as a promising therapeutic target for mitigating adipotoxicity and its associated metabolic complications.

Modulation of the microbiome–gut–adipose axis has emerged as a promising therapeutic strategy for the prevention and management of adipotoxicity and its complications. Dietary interventions rich in prebiotics and fermentable fibers, including inulin and resistant starches, promote the production of beneficial microbial metabolites such as SCFAs, which improve adipose tissue metabolism by enhancing insulin sensitivity, suppressing inflammation, and limiting ectopic lipid accumulation [11]. Supplementation with probiotic strains, particularly *Bifidobacterium* and *Lactobacillus* species, has been shown to strengthen intestinal barrier integrity, reduce circulating LPS levels, alleviate oxidative stress, and improve metabolic homeostasis [17]. In addition, polyphenol-rich foods and other plant-based natural products modulate gut microbial composition and function, increasing the abundance of beneficial microorganisms that promote adipose tissue browning, mitochondrial function, lipid oxidation, and metabolic flexibility [18,19].

Polyphenol-rich fruits have attracted considerable attention because their phytochemicals undergo extensive microbial biotransformation, generating bioactive metabolites capable of modulating glucose metabolism, lipid homeostasis, redox balance, and inflammatory signaling through the gut–adipose axis [20,21]. Among these fruits is soursop.

Soursop (*Annona muricata* L.) is a tropical fruit widely consumed for its nutritional value and long-standing use in traditional medicine. The fruit is large, heart-shaped, dark green with soft spines, typically measuring 15–20 cm in diameter, and contains creamy-white aromatic pulp surrounding numerous black seeds [22]. It is rich in flavonoids, phenolic acids, acetogenins, alkaloids, tannins, phytosterols, vitamins, and dietary fiber, many of which possess potent biological activities [23,24]. Experimental studies have demonstrated that these bioactive constituents exhibit antioxidant, anti-inflammatory, antihyperglycemic, antihypertensive, antimicrobial, and anticancer properties, although evidence from well-designed human clinical trials remains limited [23,24,25,26]. In metabolic disorders, soursop has attracted considerable attention because of its ability to modulate oxidative stress, improve insulin sensitivity, preserve pancreatic β-cell function, regulate glucose homeostasis, and attenuate inflammatory responses [24,26]. Its high dietary fiber content provides fermentable substrates for the gut microbiota, promoting the production of beneficial microbial metabolites that contribute to improved intestinal health and metabolic regulation [27,28]. Emerging evidence also suggests that polyphenols and acetogenins derived from soursop can reshape gut microbial composition and function, thereby influencing the microbiome–gut–adipose axis and its downstream effects on lipid metabolism, energy homeostasis, and adipose tissue inflammation [28,29]. These multifaceted biological activities highlight soursop as a promising functional food for the prevention and management of metabolic disorders, particularly those associated with adipotoxicity.

However, despite the growing evidence supporting the metabolic benefits of soursop, the role of the soursop–gut–adipose axis in regulating glucose homeostasis, redox balance, purinergic signaling, and lipid metabolism during adipotoxicity remains poorly understood. Therefore, the present study aimed to investigate the ability of soursop fruit and peel to enrich fecal microbial metabolites through in vitro fermentation and to evaluate the protective effects of these enriched metabolites against fructose-induced adipotoxicity. Specifically, the study investigated their modulatory effects on glucose metabolism, glutathione metabolism, carbonyl detoxification, purinergic homeostasis, inflammatory lipid signaling, and adipose tissue lipid metabolism to elucidate the mechanistic basis by which the soursop–gut–adipose axis restores metabolic homeostasis.

## 2. Materials and Methods

### 2.1. Soursop

Ripe soursop (*A. muricata*) fruits were purchased from local fruit vendors in Johannesburg, South Africa. The fruits were thoroughly washed with distilled water, after which the peels were manually separated from the pulp and the seeds removed. The fruit pulp (SWSF) and peels (SWSFP) were processed separately by homogenization with distilled water (1:0.5, *w*/*v*) using a stainless-steel blender. The resulting homogenates were centrifuged at 10,000× *g* for 10 min at 4 °C, and the supernatants were collected, freeze-dried, and stored in labeled airtight glass vials at 3 °C until further analysis.

### 2.2. Phytochemical Properties of Soursop Fruit Pulp and Peel

The phytochemical profiles of SWSF and SWSFP were characterized by gas chromatography–mass spectrometry (GC–MS) according to a previously described method [30] with slight modifications. Briefly, the samples were analyzed using an Agilent 6890 Series gas chromatograph coupled to an Agilent 5973 Mass Selective Detector (Agilent Technologies, Santa Clara, CA, USA) and controlled by Agilent ChemStation software. Compound separation was achieved on an HP-5MS capillary column (30 m × 0.25 mm internal diameter, 0.25 µm film thickness) (Agilent Technologies, Santa Clara, CA, USA) coated with (5–phenyl)-methylpolysiloxane. An aliquot (1 μL) of each sample was injected in splitless mode using ultra-high-purity helium as the carrier gas at a flow rate of 60 mL h^−1^. The initial oven temperature was maintained at 60 °C for 2 min, then increased to 285 °C at 5 °C min^−1^ and held for 3 min. The ion source and quadrupole temperatures were maintained at 230 °C and 150 °C, respectively. Mass spectra were acquired under electron ionization (EI) mode at 70 eV with an electron multiplier voltage of 1859 V. Compounds were identified by comparing their mass spectra with those contained in the National Institute of Standards and Technology (NIST) mass spectral library, 2020 release (NIST20).

### 2.3. Animals

Fifteen healthy male Sprague–Dawley rats (6–7 weeks old; 180–200 g) were obtained from the Biomedical Research Unit (BRU), University of KwaZulu-Natal, Durban, South Africa. The animals were acclimatized for seven days under standard laboratory conditions, with free access to commercial pelletized chow and water (ad libitum) and maintained on a natural 12 h light/dark photoperiod. Following acclimatization, the rats were individually housed in metabolic cages for fecal collection. Fresh fecal samples were harvested, pooled to minimize inter-individual variation in microbial composition, and immediately used for in vitro incubation with the soursop fruit and peel extracts. All experimental procedures were conducted in accordance with institutional guidelines for the care and use of laboratory animals and were approved by the Animal Ethics Committee, University of KwaZulu-Natal, Durban, South Africa (Ethical Approval Number: AREC/00002347/2021).

### 2.4. In Vitro Fecal Fermentation

The effects of soursop fruit- and peel-derived gut metabolites were evaluated using a modified method described previously [31]. Briefly, 0.5 g of freshly collected pooled fecal sample was suspended in 8 mL of sterile Krebs buffer and incubated with SWSF or SWSFP at final concentrations of 30, 60, 120, and 240 µg/mL. The incubation was carried out for 48 h at 37 °C under an atmosphere of 5% CO_2_ and 95% O_2_ to facilitate microbial fermentation. Gallic acid (30–240 µg/mL) was included as a reference antioxidant and subjected to identical incubation conditions. An unfermented control, consisting of freshly collected fecal samples without incubation or treatment, was included for comparison. Following incubation, the fermentation mixtures were homogenized and centrifuged at 20,000× *g* for 10 min at 4 °C. The resulting supernatants, representing the gut microbiota-derived metabolites, were carefully collected into sterile 2 mL microcentrifuge tubes and stored at −80 °C until subsequent biochemical, metabolomic, and enzymatic analyses.

### 2.5. Metabolic Profiling of Fecal Microbiota

Fecal metabolites were extracted according to a previously described method [32] with slight modifications. Briefly, 1000 μL of fecal supernatant was mixed with an equal volume of methanol/ethanol (2:8, *v*/*v*) to precipitate proteins and incubated on ice for 20 min. The mixture was centrifuged at 20,000× *g* for 10 min at 4 °C, and the resulting supernatant was transferred into HPLC vials. The extracted fecal metabolites were profiled by Gas Chemical–Mass Spectroscopy (GC–MS) using an Agilent 6890 Series gas chromatograph coupled to an Agilent 5973 Mass Selective Detector (Agilent Technologies, Santa Clara, CA, USA) and controlled by Agilent ChemStation software (version E.02.02.1431; Agilent Technologies, Santa Clara, CA, USA). Metabolite separation was achieved on an HP-5MS capillary column. An aliquot (1 μL) of each extract was injected in splitless mode using ultra-high-purity helium as the carrier gas at a flow rate of 60 mL h^−1^. The initial oven temperature was maintained at 60 °C for 2 min, increased to 285 °C at a rate of 5 °C min^−1^, and held for 3 min. The ion source and quadrupole temperatures were maintained at 230 °C and 150 °C, respectively. Mass spectra were acquired under electron ionization mode at 70 eV with an electron multiplier voltage of 1859 V. Metabolites were identified by comparing their mass spectra with entries in the National Institute of Standards and Technology mass spectral library.

#### Metabolomics Analysis

The identified metabolites were subjected to omics analyses using the MetaboAnalyst 6.0 online server (https://www.metaboanalyst.ca/ accessed on 24 February 2024) [33].

### 2.6. Collection of Adipose Tissues

Prior to tissue collection, the rats were fasted overnight (8 h) with free access to water. The animals were then humanely euthanized under isoflurane anesthesia, and blood samples were immediately collected by cardiac puncture. Following euthanasia, the abdominal cavity was opened, and perigonadal white adipose tissue (pgWAT) was carefully excised, rinsed in ice-cold physiological saline to remove residual blood, blotted dry, and immediately processed for induction of adipotoxicity and subsequent biochemical analyses.

### 2.7. Induction and Treatment of Adipotoxicity

Ex vivo adipotoxicity was induced by incubating 0.5 g of freshly isolated pgWAT in 8 mL of Krebs buffer supplemented with 25 mM fructose [34] for 48 h at 37 °C under an atmosphere of 5% CO_2_ and 95% O_2_. This fructose-treated group served as the negative control. Treatment groups consisted of pgWAT co-incubated with 25 mM fructose and SWSF-, SWSFP-, or gallic acid-enriched fecal metabolites at final concentrations of 30, 60, 120, and 240 µg/mL under the same incubation conditions. A normal control comprised freshly isolated pgWAT that was not subjected to ex vivo incubation or treatment.

Following incubation, the adipose tissues were homogenized in ice-cold 50 mM phosphate buffer (pH 7.5) containing 1% Triton X-100 (Sigma-Aldrich, Darmstadt, Germany). The homogenates were centrifuged at 20,000× *g* for 10 min at 4 °C, and the resulting supernatants were carefully collected into sterile 2 mL microcentrifuge tubes and stored at −80 °C until subsequent biochemical, enzymatic, and metabolomic analyses.

### 2.8. Glucose Metabolism Enzyme Activities

The activities of key enzymes involved in glucose metabolism, including fructose-1,6-bisphosphatase (FBPase), glucose-6-phosphatase (G6Pase), and glycogen phosphorylase (GP), were determined in adipose tissue homogenates according to previously described methods [35,36].

#### 2.8.1. Fructose-1,6-Bisphosphatase Activity

Briefly, 100 μL of adipose tissue supernatant was incubated with 100 μL of 0.05 M fructose-1,6-bisphosphate, 1.2 mL of 0.1 M Tris–HCl buffer (pH 7.0), 250 μL of 0.1 M MgCl_2_, 100 μL of 0.1 M KCl, and 250 μL of 1 mM EDTA at 37 °C for 15 min. The reaction was terminated by the addition of 10% (*w*/*v*) trichloroacetic acid (TCA), followed by centrifugation at 3000× *g* for 10 min at 4 °C. An aliquot (100 μL) of the resulting supernatant was transferred to a 96-well microplate, followed by the addition of 50 μL each of freshly prepared 9% (*w*/*v*) ascorbic acid and 1.25% (*w*/*v*) ammonium molybdate. After incubation at room temperature for 20 min, the liberated inorganic phosphate (Pi) was quantified by measuring absorbance at 680 nm using a SpectraMax M2 microplate reader (Molecular Devices, San Jose, CA, USA). FBPase activity was calculated from a sodium phosphate standard curve and expressed as μmol Pi released min^−1^.

#### 2.8.2. Glucose 6-Phosphatase Activity

Briefly, 200 μL of adipose tissue supernatant was incubated with 100 μL of 0.25 M glucose-6-phosphate, 200 μL of 5 mM KCl, and 1.3 mL of 0.1 M Tris–HCl buffer (pH 7.0) at 37 °C for 30 min in a shaking incubator. The reaction was terminated by the addition of 1 mL distilled water followed by 1 mL of 1.25% (*w*/*v*) ammonium molybdate. Subsequently, 1 mL of freshly prepared 9% (*w*/*v*) ascorbic acid was added, and the reaction mixture was incubated at room temperature for 30 min to allow color development. The liberated inorganic phosphate (Pi) was quantified by measuring absorbance at 660 nm using a SpectraMax M2 microplate reader (Molecular Devices, San Jose, CA, USA). G6Pase activity was calculated from a sodium phosphate standard curve and expressed as μmol Pi released min^−1^.

#### 2.8.3. Glycogen Phosphorylase Activity

Briefly, 100 μL of adipose tissue supernatant was incubated with 64 mM glucose-1-phosphate and 4% (*w*/*v*) glycogen at 37 °C for 10 min. The reaction was terminated by the addition of 20% (*w*/*v*) ammonium molybdate prepared in concentrated sulfuric acid (H_2_SO_4_). Subsequently, Elon reducing reagent and distilled water were added, and the reaction mixture was incubated at 30 °C for 45 min to allow color development. The liberated inorganic phosphate (Pi) was quantified by measuring absorbance at 660 nm using a SpectraMax M2 microplate reader (Molecular Devices, San Jose, CA, USA). Glycogen phosphorylase activity was calculated from a sodium phosphate standard curve and expressed as μmol Pi released min^−1^.

### 2.9. Polyol Pathway Enzyme Activities

Polyol pathway activity was assessed by determining the activities of its two key enzymes, aldose reductase and sorbitol dehydrogenase, in adipose tissue homogenates using previously described methods [37,38].

#### 2.9.1. Aldose Reductase Activity

Briefly, 100 μL of adipose tissue supernatant was added to a reaction mixture containing 700 μL of phosphate buffer (pH 6.7), 100 μL of 0.25 mM nicotinamide adenine dinucleotide phosphate (NADPH), and 100 μL of DL-glyceraldehyde as the substrate. The reaction was carried out at 25 °C, and the oxidation of NADPH was monitored by measuring the decrease in absorbance at 340 nm for 3 min at 30 s intervals using a SpectraMax M2 microplate reader (Molecular Devices, San Jose, CA, USA). Aldose reductase activity was calculated from the rate of NADPH consumption using the molar extinction coefficient of NADPH (6.22 mM^−1^ cm^−1^).

#### 2.9.2. Sorbitol Dehydrogenase Activity

Briefly, 10 μL of adipose tissue supernatant was added to 240 μL of 50 mM glycine–NaOH buffer (pH 10.0) containing 57 mM sorbitol in a 96-well microplate. The reaction was initiated by adding 50 μL of 50 mM NAD^+^ and incubated at 25 °C. The reduction of NAD^+^ to NADH was monitored by measuring the increase in absorbance at 340 nm for 3–5 min at 30 s intervals using a SpectraMax M2 microplate reader (Molecular Devices, San Jose, CA, USA). Sorbitol dehydrogenase activity was calculated from the rate of NADH formation using the molar extinction coefficient of NADH (6.22 mM^−1^ cm^−1^).

### 2.10. Glutathione Metabolism

The status of glutathione metabolism was evaluated by measuring reduced glutathione (GSH) levels and the activities of glutathione reductase (GR) and glutathione peroxidase (GPx) in adipose tissue homogenates using previously described methods [39,40,41].

#### 2.10.1. Glutathione Peroxidase Activity

Briefly, 5 μL of adipose tissue supernatant was incubated with 210 μL of phosphate buffer (pH 6.9), 2.5 μL of 100 mM reduced glutathione (GSH), and 5 μL of distilled water in a 96-well microplate at 37 °C for 10 min. Subsequently, 2.5 μL of 15 mM NADPH and 10 μL of 1 mM 5,5′-dithiobis-(2-nitrobenzoic acid) (DTNB) were added to the reaction mixture. Absorbance was measured at 412 nm using a SpectraMax M2 microplate reader (Molecular Devices, San Jose, CA, USA). GPx activity was calculated from a GSH standard curve.

#### 2.10.2. Glutathione Reductase Activity

Briefly, 10 μL of adipose tissue supernatant was mixed with 221 μL of 50 mM Tris–HCl buffer (pH 8.0) containing 1 mM EDTA and 38 μL of 8 mM oxidized glutathione (GSSG) in a 96-well microplate. The reaction was initiated by the addition of 10 μL of nicotinamide adenine dinucleotide phosphate (NADPH) and monitored by measuring the decrease in absorbance at 340 nm every 2 min for 8 min using a SpectraMax M2 microplate reader (Molecular Devices, San Jose, CA, USA). Glutathione reductase activity was calculated from the rate of NADPH oxidation using the molar extinction coefficient of NADPH (6.22 mM^−1^ cm^−1^).

#### 2.10.3. GSH Level

Briefly, 150 μL of adipose tissue supernatant was mixed with an equal volume of 10% (*w*/*v*) trichloroacetic acid (TCA) and centrifuged at 2000× *g* for 10 min at room temperature. An aliquot (80 μL) of the resulting supernatant was transferred to a 96-well microplate and mixed with 40 μL of 0.5 mM 5,5′-dithiobis-(2-nitrobenzoic acid) (DTNB). Subsequently, 200 μL of 0.2 M phosphate buffer (pH 7.8) was added, and the reaction mixture was incubated at room temperature for 15 min. Absorbance was measured at 415 nm using a SpectraMax M2 microplate reader (Molecular Devices, San Jose, CA, USA). GSH levels were quantified from a GSH standard curve.

### 2.11. Glyoxalase-1 Activity

Glyoxalase-1 (GLO-1) activity was determined according to a previously described method [42] with slight modifications. Briefly, 50 μL of 50 mM phosphate buffer (pH 6.6) was mixed with equal volumes of 2 mM methylglyoxal and 2 mM reduced glutathione (GSH) and incubated at 37 °C for 30 min to allow the formation of the hemithioacetal substrate. Subsequently, 10 μL of adipose tissue supernatant was added, and the reaction mixture was further incubated at 37 °C for 10 min. Glyoxalase-1 activity was monitored by measuring the increase in absorbance associated with the formation of S-D-lactoylglutathione at 240 nm. Absorbance was recorded every 2 min for 8 min using a SpectraMax M2 microplate reader (Molecular Devices, San Jose, CA, USA). GLO-1 activity was calculated from the rate of S-D-lactoylglutathione formation using the molar extinction coefficient of 2.86 mM^−1^ cm^−1^.

### 2.12. Nucleotide Metabolism

Nucleotide metabolism was assessed by determining the activities of the purinergic enzymes, adenosine triphosphatase (ATPase) and ecto-nucleoside triphosphate diphosphohydrolase (ENTPDase) in adipose tissue homogenates according to previously described methods [43,44].

#### 2.12.1. ATPase Activity

Briefly, 200 μL of adipose tissue supernatant was incubated with 200 μL of 5 mM KCl, 1.3 mL of 0.1 M Tris–HCl buffer (pH 7.0), and 40 μL of 50 mM adenosine triphosphate (ATP) at 37 °C for 30 min in a shaking incubator. The reaction was terminated by the addition of 1 mL distilled water followed by 1 mL of ammonium molybdate. Subsequently, 1 mL of freshly prepared 9% (*w*/*v*) ascorbic acid was added, and the reaction mixture was incubated on ice for 10 min to allow color development. The liberated inorganic phosphate (Pi) was quantified by measuring absorbance at 660 nm using a SpectraMax M2 microplate reader (Molecular Devices, San Jose, CA, USA). ATPase activity was calculated from a sodium phosphate standard curve and expressed as μmol Pi released min^−1^.

#### 2.12.2. E-NTPDase Activity

Briefly, 20 μL of adipose tissue supernatant was incubated with 200 μL of reaction buffer containing 1.5 mM CaCl_2_, 5 mM KCl, 0.1 mM EDTA, 10 mM glucose, 225 mM sucrose, and 45 mM Tris–HCl buffer (pH 7.4) at 37 °C for 10 min. The reaction was initiated by adding 20 μL of 50 mM adenosine triphosphate (ATP) and further incubated at 37 °C for 20 min in a shaking incubator. The reaction was terminated by the addition of 200 μL of 10% (*w*/*v*) trichloroacetic acid (TCA). Subsequently, 200 μL of 1.25% (*w*/*v*) ammonium molybdate and 200 μL of freshly prepared 9% (*w*/*v*) ascorbic acid were added for color development. The reaction mixture was incubated on ice for 10 min, after which absorbance was measured at 600 nm using a SpectraMax M2 microplate reader (Molecular Devices, San Jose, CA, USA). E-NTPDase activity was calculated from a sodium phosphate standard curve and expressed as μmol Pi released min^−1^.

### 2.13. Lipoxygenase Activities

Lipoxygenase (5-LOX and 12/15-LOX) activities were determined according to a previously described method [45] with slight modifications. Briefly, 20 μL of adipose tissue supernatant was incubated with 100 μL of lipoxygenase (LOX) reaction buffer for 5 min at room temperature. For the 5-LOX assay, the reaction buffer consisted of 50 mM Tris–HCl (pH 7.5), 0.3 mM CaCl_2_, 0.1 mM EDTA, and 0.1 mM adenosine triphosphate (ATP), whereas the 12/15-LOX assay contained only 50 mM Tris–HCl (pH 7.5). The reaction was initiated by adding 40 μL of linolenic acid as the substrate. Lipoxygenase activity was monitored by measuring the increase in absorbance at 234 nm, with readings recorded every 30 s for 2 min using a SpectraMax M2 microplate reader (Molecular Devices, San Jose, CA, USA).

### 2.14. Metabolic Profiling of Adipose Tissues

Adipose metabolites were extracted according to a previously described method [32] with slight modifications. Briefly, 1000 μL of adipose tissue supernatant was mixed with an equal volume of methanol/ethanol (2:8, *v*/*v*) to precipitate proteins and incubated on ice for 20 min. The mixture was centrifuged at 20,000× *g* for 10 min at 4 °C, and the resulting supernatant was transferred into HPLC vials. The extracted adipose metabolites were profiled by GC–MS using an Agilent 6890 Series gas chromatograph coupled to an Agilent 5973 Mass Selective Detector (Agilent Technologies, Santa Clara, CA, USA) and controlled by Agilent ChemStation software. Metabolite separation was achieved on an HP-5MS capillary column (30 m × 0.25 mm internal diameter, 0.25 µm film thickness) coated with (5–phenyl)-methylpolysiloxane. An aliquot (1 μL) of each extract was injected in splitless mode using ultra-high-purity helium as the carrier gas at a flow rate of 60 mL h^−1^. The initial oven temperature was maintained at 60 °C for 2 min, followed by a programmed increase to 285 °C at a rate of 5 °C min^−1^ and held for 3 min. The ion source and quadrupole temperatures were maintained at 230 °C and 150 °C, respectively. Mass spectra were acquired under electron ionization (EI) mode at 70 eV with an electron multiplier voltage of 1859 V. Metabolites were identified by comparing their mass spectra with entries in the National Institute of Standards and Technology (NIST) mass spectral library.

#### Metabolomics Analysis

The identified metabolites were subjected to multivariate and pathway analyses using the MetaboAnalyst 6.0 online platform (https://www.metaboanalyst.ca/ accessed on 24 February 2024) [33]. Prior to analysis, metabolite data were normalized and autoscaled. Principal component analysis (PCA), partial least squares-discriminant analysis (PLS-DA), variable importance in projection (VIP) scores, heatmaps, and metabolic pathway enrichment analyses were performed to identify treatment-induced metabolic alterations.

### 2.15. AI-Assisted Schematic Illustration

OpenAI’s ChatGPT (GPT-5.6-sol model with extra-high reasoning effort; OpenAI, San Francisco, CA, USA) was used solely to assist in generating the conceptual schematic, illustrating the proposed biochemical pathways based on the authors’ scientific interpretation of results from the study. The generated schematic was subsequently reviewed, refined, and approved by the authors to ensure scientific accuracy.

## 3. Statistical Analysis

All experiments were performed in biological triplicate, and the results are presented as the mean ± standard deviation (SD). Statistical analyses were performed using two-way analysis of variance (ANOVA) with IBM SPSS Statistics version 27.0 (IBM Corp., Armonk, NY, USA). Differences were considered statistically significant at *p* < 0.05 following Tukey’s HSD multiple range post hoc test.

## 4. Results and Discussion

Adipotoxicity is a key contributor to the pathogenesis and progression of metabolic dysfunction, including obesity, insulin resistance, and type 2 diabetes [1,2]. The modulatory effects of natural products on the gut–adipose axis have attracted increasing attention as a promising therapeutic strategy for preventing and ameliorating adipose tissue dysfunction and its associated metabolic complications [46,47]. In the present study, we investigated the protective role of the soursop–gut–adipose axis in attenuating fructose-induced adipotoxicity by evaluating ex vivo gut microbial metabolism and its downstream effects on adipose glucose and lipid metabolism, redox homeostasis, inflammatory signaling, and metabolomic profiles.

### 4.1. Soursop–Fecal Fermentation

**Figure 1:**
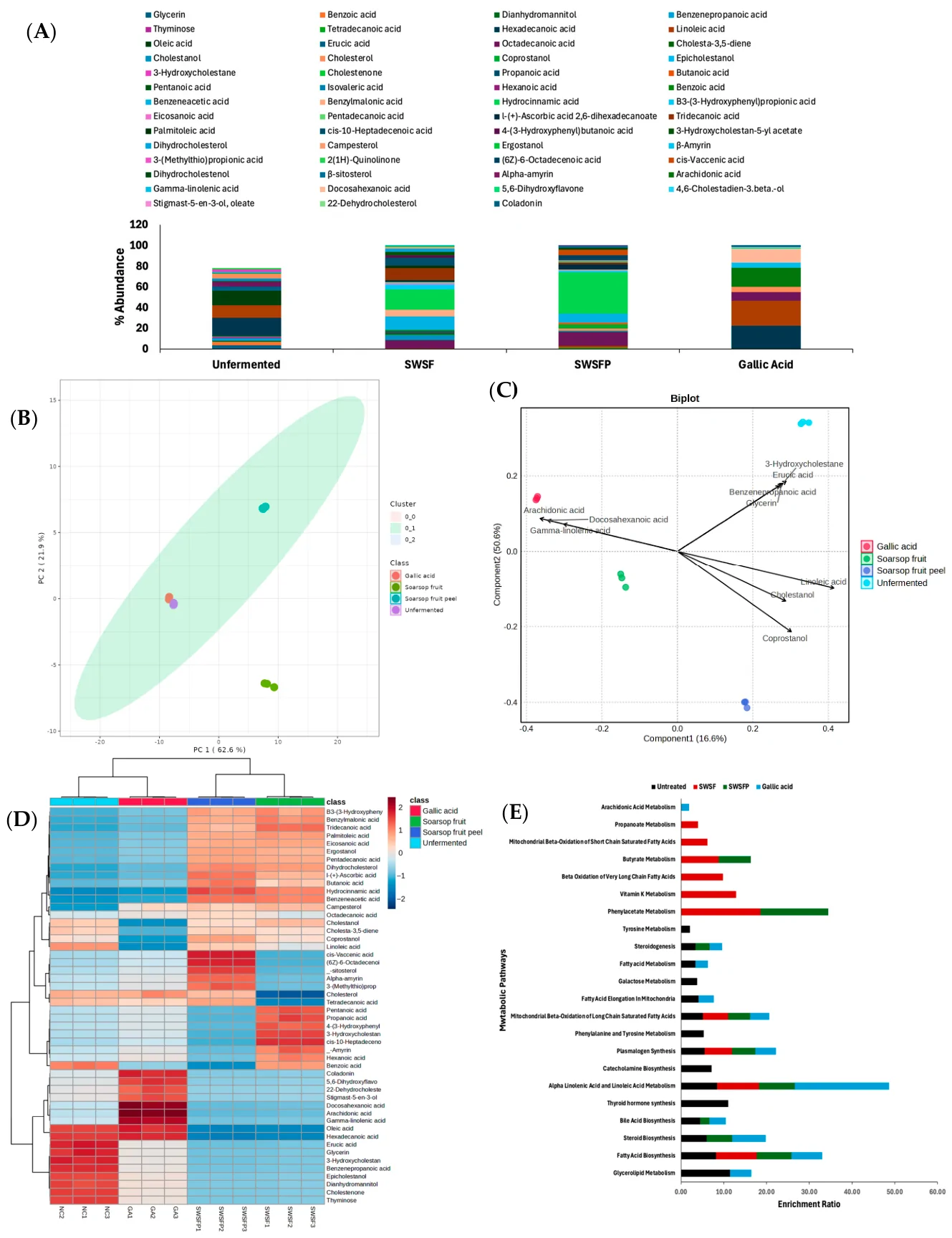
Metabolomic characterization of soursop-enriched fecal metabolites. (**A**) Relative abundance of fecal metabolites; (**B**) principal component analysis (PCA) score plot; (**C**) partial least squares (PLS) biplot; (**D**) heatmap of differentially abundant fecal metabolites; and (**E**) pathway enrichment analysis of soursop-enriched fecal metabolites. SWSF = soursop fruit; SWSFP = soursop fruit peel.

As shown in Figure 1, GC–MS metabolomic analysis revealed that fermentation with soursop led to significant alterations in the fecal metabolite profile compared with the unfermented and gallic acid groups. The relative abundance profiles revealed substantial differences in fatty acids, sterols, and microbial fermentation products, indicating that SWSF and SWSFP generated distinct metabolite profiles during fermentation (Figure 1A).

Principal component analysis (Figure 1B) clearly discriminated the treatment groups, with PC1 and PC2 accounting for 62.6% and 21.9% of the total variance, respectively. SWSF and SWSFP formed distinct clusters, separate from the unfermented and gallic acid groups, indicating that the fruit and peel differentially modulate fecal metabolites. Consistent with the PCA, the PLS biplot identified palmitic acid, β-sitosterol, coprostanol and erucic acid as the principal metabolites associated with SWSF, whereas cholesterol, epicholestanol and 3-hydroxycholestane were more strongly associated with SWSFP (Figure 1C). The unfermented and gallic acid groups were characterized by relatively higher contributions from arachidonic acid, γ-linolenic acid and docosahexaenoic acid. Heatmap analysis further confirmed distinct metabolite signatures among the treatment groups (Figure 1D).

The differential metabolites are closely associated with glucose and lipid metabolism. β-Sitosterol and coprostanol have been reported to improve cholesterol turnover and lipid homeostasis, thereby limiting lipid accumulation and improving insulin sensitivity [48]. Similarly, alterations in cholesterol and its derivatives, including epicholestanol and 3-hydroxycholestane, suggest modulation of sterol metabolism, which is essential for membrane integrity, insulin receptor function, and cellular lipid balance [48,49]. The association of palmitic acid with SWSF indicates remodeling of fatty acid metabolism, while its concurrent enrichment within fatty acid biosynthesis and β-oxidation pathways suggests enhanced fatty acid turnover rather than simple lipid accumulation [50,51]. Furthermore, the differential abundance of arachidonic acid and γ-linolenic acid, both key mediators of polyunsaturated fatty acid metabolism, may influence inflammatory lipid signaling and insulin responsiveness [52], while docosahexaenoic acid has been linked with improved membrane fluidity and glucose utilization [53].

As shown in Figure 1E, pathway enrichment analysis demonstrated that the altered metabolites were predominantly involved in fatty acid biosynthesis, fatty acid metabolism, mitochondrial β-oxidation of long-chain fatty acids, glycerolipid metabolism, plasmalogen synthesis, α-linolenic and linoleic acid metabolism, arachidonic acid metabolism, steroid biosynthesis and bile acid biosynthesis. These pathways collectively regulate fatty acid synthesis, lipid oxidation, membrane lipid remodeling and sterol metabolism, all of which are closely linked to insulin sensitivity and glucose homeostasis. The enrichment of β-oxidation and fatty acid metabolic pathways suggests improved lipid utilization, while modulation of glycerolipid and plasmalogen metabolism may preserve membrane integrity and adipocyte function [54]. Similarly, changes in sterol- and bile acid-related pathways may contribute to improved cholesterol metabolism and metabolic signaling. These results suggest that soursop-fecal fermentation generates metabolites that remodel metabolic pathways involved in glucose and lipid homeostasis, thereby providing a mechanistic basis for the subsequent attenuation of fructose-induced adipotoxicity.

### 4.2. Phytochemical Profile of Soursop

GC–MS analysis revealed distinct phytochemical profiles for SWSF and SWSFP, with several compounds common to both extracts (Table 1). The predominant constituent of SWSF was 3,5-dihydroxy-6-methyl-2,3-dihydro-4H-pyran-4-one (35%), followed by acetic acid (24%), decahydroquinoline-10-ol (20%), and 3,7-dimethylimidazo [1,2-a]pyrimidine-2,5(1H,3H)-dione (10%), while SWSFP was characterized by decahydroquinolin-10-ol (30.36%), 3,5-dihydroxy-6-methyl-2,3-dihydro-4H-pyran-4-one (24%), octahydro-2(1H)-quinolinone (21.38%), and lower proportions of 6-octadecenoic acid, L-(+)-ascorbic acid 2,6-dihexadecanoate, 13-docosenamide, octadecanoic acid, glyceryl monooleate, glycerol 1-palmitate, and γ-sitosterol.

The presence of 3,5-dihydroxy-6-methyl-2,3-dihydro-4H-pyran-4-one in both extracts is noteworthy because this Maillard reaction-derived compound has been reported for its antioxidant activity [55], suggesting that both the fruit and peel may contribute to protection against oxidative stress. The presence of L-(+)-ascorbic acid 2,6-dihexadecanoate further corroborates the antioxidant potential of both extracts [56]. The peel contained several lipid-associated compounds, including γ-sitosterol, glycerol 1-palmitate, glyceryl monooleate, and octadecanoic acid, which have been associated with cholesterol homeostasis, lipid metabolism, membrane stability and anti-inflammatory activities [57,58], thus suggesting that the peel may possess a higher potential for modulation of lipid metabolic processes.

The fruit extract possessed appreciable amounts of acetic acid and decahydroquinoline-10-ol, together with 6-methylpteridine-2,4,7-trione, indicating a phytochemical composition enriched in small organic acids and nitrogen-containing heterocyclic compounds. Acetic acid is a precursor of acetyl-CoA and has been implicated in the regulation of glucose and lipid metabolism [59], while quinoline and pteridine derivatives have been reported to exhibit antioxidant and bioactive properties [60,61]. Although the biological activities of some of these compounds remain incompletely characterized, their differential abundance suggests that the fruit and peel provide chemically distinct substrates for subsequent fermentation.

These results demonstrate that SWSF and SWSFP possess distinct yet complementary phytochemical compositions which may contribute to the differential metabolic outcomes observed following fecal fermentation and support the subsequent evaluation of soursop-enriched fermentation metabolites in fructose-induced adipotoxicity.

### 4.3. Glucose Metabolism

**Figure 2:**
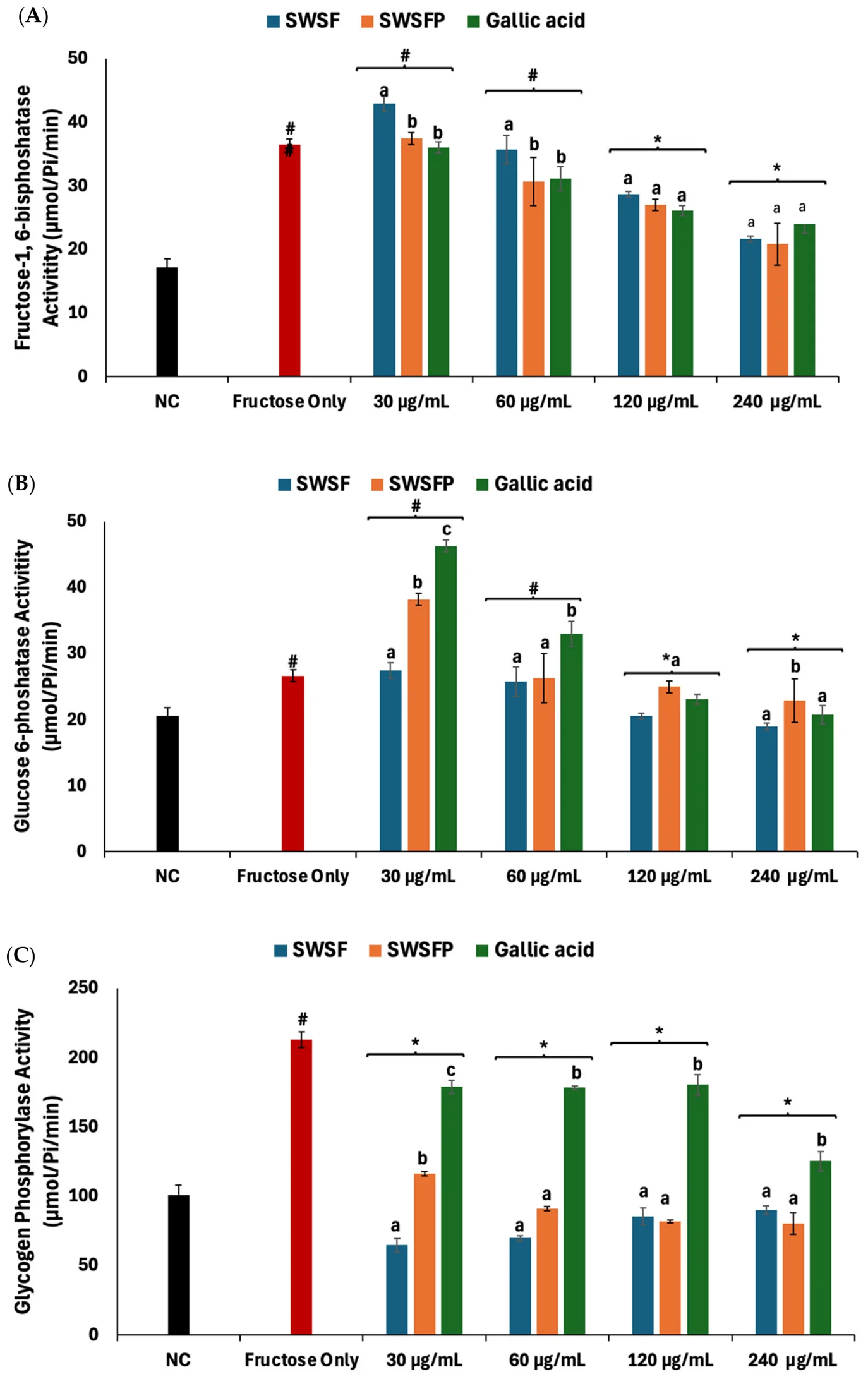
Effect of soursop-enriched fecal metabolites on (**A**) fructose-1,6-bisphosphatse; (**B**) glucose 6-phosphatase; and (**C**) glycogen phosphorylase activities in fructose-induced adipotoxicity. Data = mean ± SD; n = 3. * Statistically significant (*p* < 0.05) compared to fructose only; # statistically significant (*p* < 0.05) compared to NC. ^a,b,c^ Values with different letters above the bars are significantly (*p* < 0.05) different from each other. NC = normal control; SWSF = soursop fruit; SWSFP = soursop fruit peel.

As shown in Figure 2, fructose-induced adipotoxicity significantly (*p* < 0.05) elevated the activities of F-1,6-BPase, G6Pase, and glycogen phosphorylase compared with the normal control, indicating profound disturbances in intracellular glucose metabolism. Treatment with soursop-enriched fecal metabolites significantly (*p* < 0.05) attenuated the activities of all three enzymes in a dose-dependent manner, thereby restoring glucose homeostasis.

F-1,6-BPase activity was significantly (*p* < 0.05) elevated following fructose exposure but was significantly depleted by SWSF, SWSFP, and gallic acid, with SWSF producing the greatest inhibition at higher concentrations (Figure 2A). F-1,6-BPase is a key regulatory enzyme of gluconeogenesis, and its aberrant activation in adipocytes reflects impaired insulin responsiveness and metabolic inflexibility [62]. Although adipose tissue is not a major gluconeogenic organ, increased expression and/or activity of gluconeogenic enzymes under obesogenic conditions has been associated with dysfunctional glucose cycling, adipocyte hypertrophy and impaired insulin signaling [63]. Thus, suppression of F-1,6-BPase suggests restoration of metabolic flexibility and improved glucose utilization.

Similarly, fructose significantly (*p* < 0.05) elevated G6Pase activity, whereas treatment with the soursop-enriched fecal metabolites significantly suppressed the enzyme (Figure 2B). G6Pase catalyzes the final step of gluconeogenesis and glycogenolysis, and excessive activity reduces intracellular glucose-6-phosphate availability for glycolysis, glycogen synthesis and the pentose phosphate pathway. Thus, elevated G6Pase activity limits ATP production and NADPH generation, thereby predisposing adipocytes to oxidative stress and insulin resistance [64]. Inhibition of G6Pase therefore indicates improved intracellular glucose retention and enhanced metabolic homeostasis.

Fructose also significantly (*p* < 0.05) stimulated glycogen phosphorylase activity (Figure 2C), indicating accelerated glycogen degradation (glycogenolysis). SWSF, SWSFP and gallic acid significantly reduced the enzyme activity, with SWSF exhibiting greater inhibition at higher concentrations. Glycogen phosphorylase catalyzes the rate-limiting step of glycogenolysis; thus, its suppression favors glycogen conservation and prevents excessive mobilization of intracellular glucose reserves [65], thereby contributing to improved glucose handling.

These beneficial effects are consistent with the metabolomic alterations observed in Figure 1, where fermentation with soursop generated metabolites enriched in pathways related to fatty acid metabolism, glycerolipid metabolism, sterol metabolism, β-oxidation and polyunsaturated fatty acid metabolism. Fermentation-derived metabolites such as SCFAs, sterol derivatives and bioactive lipids are reported mediators of the gut–adipose axis, acting as signaling molecules that regulate adipocyte metabolism through insulin-dependent and insulin-independent mechanisms [66]. By improving gut-derived metabolite composition, soursop fermentation may enhance adipocyte metabolic flexibility, suppress inappropriate activation of gluconeogenic enzymes and reduce metabolic stress associated with fructose overload. The coordinated inhibition of F-1,6-BPase, G6Pase and glycogen phosphorylase therefore suggests that soursop-enriched fecal metabolites remodeled adipocyte glucose metabolism through the gut–adipose metabolic axis, favoring intracellular glucose utilization over excessive glucose production and glycogen degradation. This metabolic shift could reduce substrate oversupply to lipogenic pathways, improve insulin sensitivity, and ultimately attenuate lipid accumulation and adipocyte dysfunction.

### 4.4. Polyol Pathway

**Figure 3:**
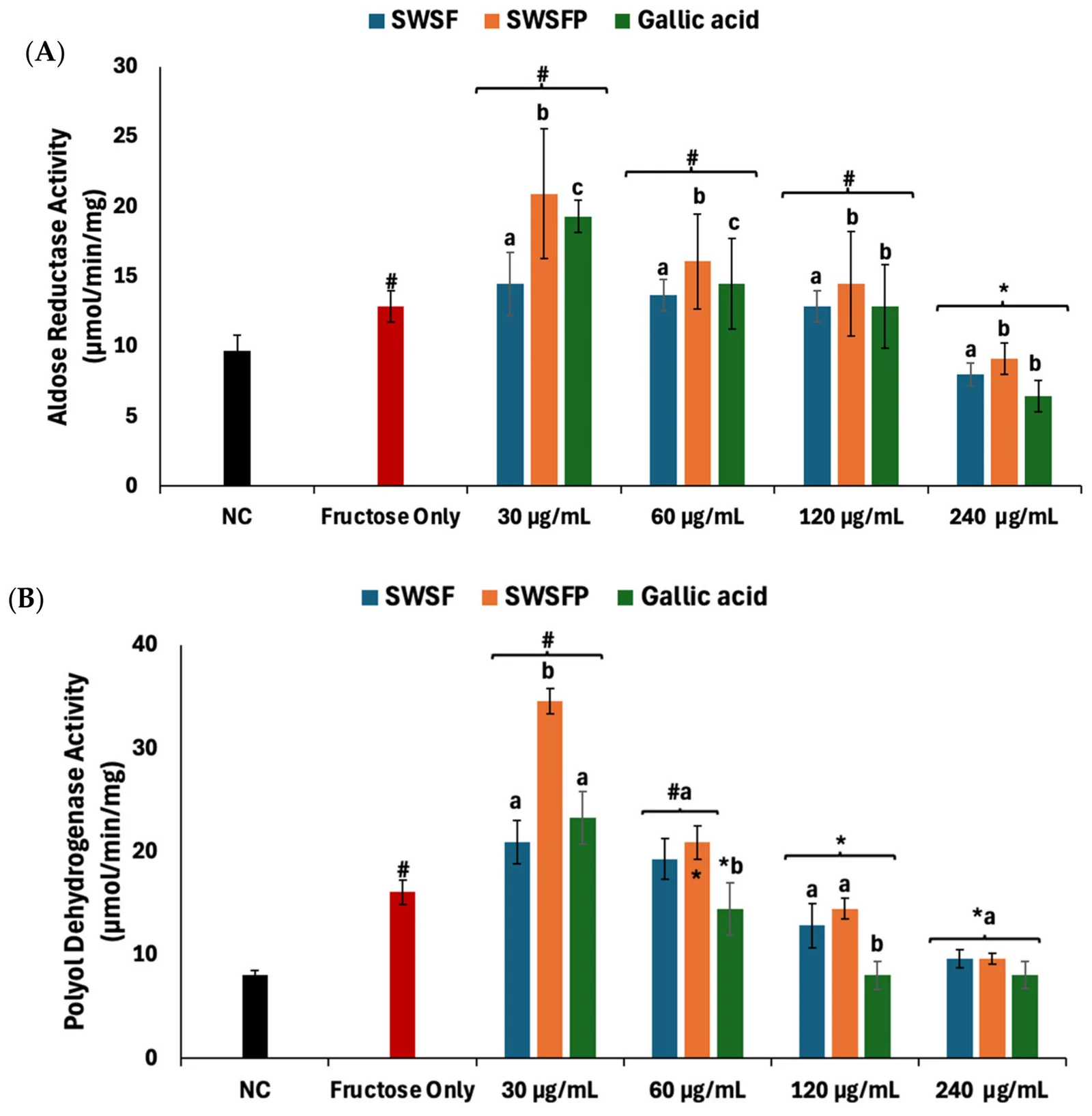
Effect of soursop-enriched fecal metabolites on (**A**) aldose reductase; and (**B**) polyol dehydrogenase activities in fructose-induced adipotoxicity. Data = mean ± SD; n = 3. * Statistically significant (*p* < 0.05) compared to fructose only; # statistically significant (*p* < 0.05) compared to NC. ^a,b,c^ Values with different letters above the bars are significantly *(p* < 0.05) different from each other. NC = normal control; SWSF = soursop fruit; SWSFP = soursop fruit peel.

Fructose-induced adipotoxicity significantly (*p* < 0.05) exacerbated the activities of aldose reductase and SDH compared with the normal control, as shown in Figure 3, indicating activation of the polyol pathway under conditions of metabolic stress. Treatment with soursop-enriched fecal metabolites significantly (*p* < 0.05) suppressed the activities of both enzymes in a dose-dependent manner.

Aldose reductase activity was significantly (*p* < 0.05) elevated following exposure to fructose but was significantly reduced by SWSF, SWSFP and gallic acid (Figure 3A). SWSF exhibited the strongest inhibition at higher concentrations, while SWSFP and gallic acid showed stronger effects at lower concentrations. AR catalyzes the reduction of glucose to sorbitol using NADPH as a cofactor. Under hyperglycemic and insulin-resistant conditions, excessive AR activity diverts glucose into the polyol pathway, resulting in a depletion in intracellular NADPH [67]. As NADPH is essential for regenerating GSH, sustained activation of AR compromises antioxidant defenses, enhances reactive oxygen species production and promotes oxidative damage within adipocytes [68]. Thus, inhibition of AR preserves cellular reducing capacity and limits oxidative stress associated with fructose-induced adipotoxicity.

Similarly, fructose significantly (*p* < 0.05) exacerbated SDH activity, while treatment with soursop-enriched fecal metabolites effectively attenuated the enzyme activity (Figure 3B). SDH catalyzes the oxidation of sorbitol to fructose, generating NADH and thereby increasing the intracellular NADH/NAD^+^ ratio. Excessive SDH activity promotes reductive stress, mitochondrial dysfunction and enhanced formation of reactive carbonyl species [69], further aggravating insulin resistance and lipid accumulation. Therefore, concomitant inhibition of AR and PDH interrupts excessive polyol flux, reducing fructose generation and preventing amplification of oxidative and carbonyl stress.

These results provide important insights for the gut–adipose axis proposed in the present study. As demonstrated in Figure 1, fermentation of soursop generated metabolites enriched in pathways associated with fatty acid metabolism, glycerolipid metabolism and sterol metabolism. By suppressing activation of the polyol pathway, the soursop-enriched fecal metabolites reduce NADPH depletion, preserve intracellular antioxidant capacity and limit fructose-driven carbonyl stress, thereby preventing the metabolic conditions that initiate adipocyte dysfunction. This coordinated inhibition further complements the suppression of glucogenic enzymes observed in Figure 2, thus suggesting that soursop-mediated modulation of gut-derived metabolites attenuates fructose-induced adipotoxicity by simultaneously improving glucose metabolism and limiting polyol pathway activation.

### 4.5. Glutathione Metabolism

**Figure 4:**
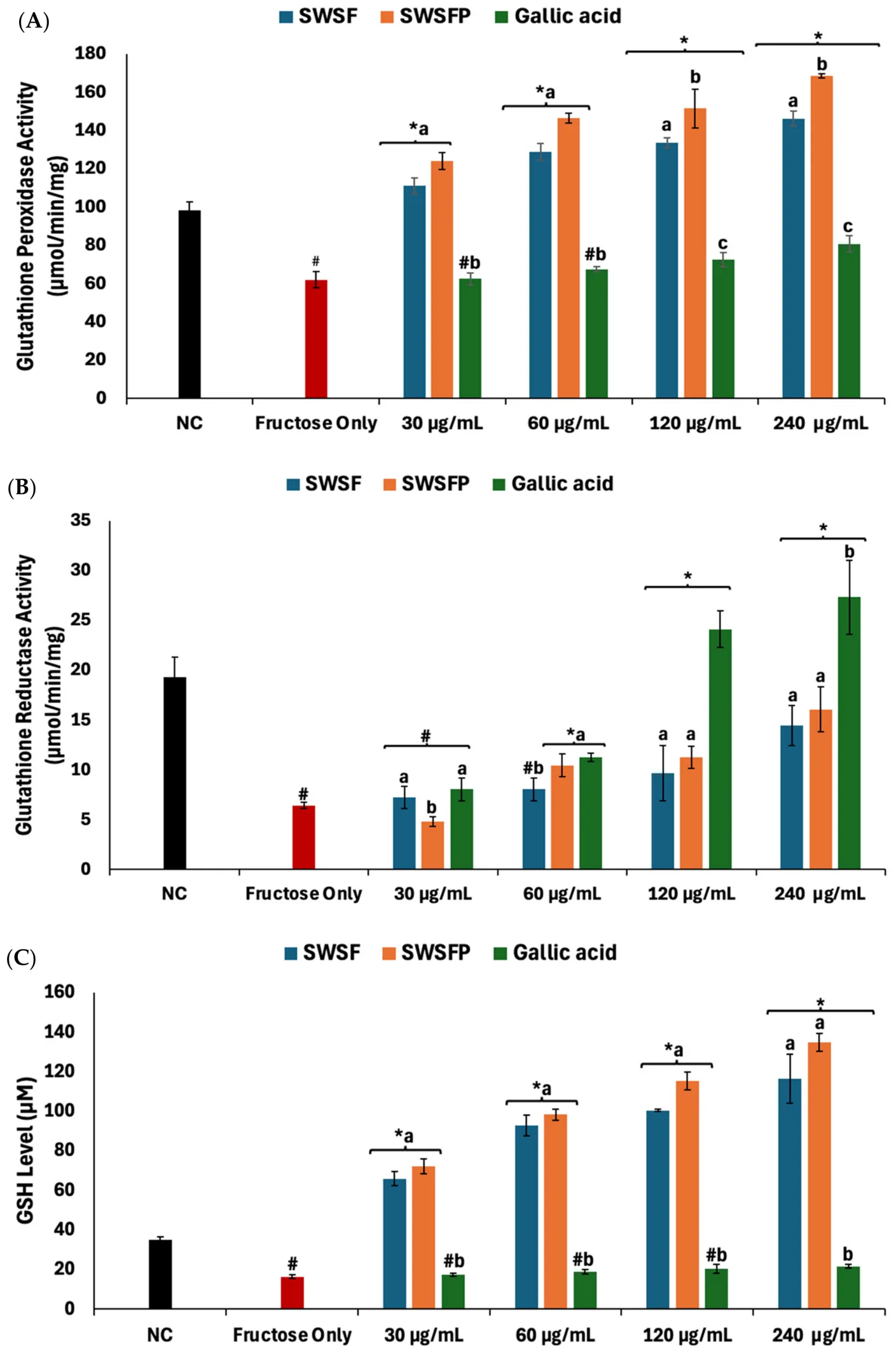
Effect of soursop-enriched fecal metabolites on (**A**) glutathione peroxidase; (**B**) glutathione reductase activities; and (**C**) GSH level in fructose-induced adipotoxicity. Data = mean ± SD; n = 3. * Statistically significant (*p* < 0.05) compared to fructose only; # statistically significant (*p* < 0.05) compared to NC. ^a,b,c^ Values with different letters above the bars are significantly (*p* < 0.05) different from each other. NC = normal control; SWSF = soursop fruit; SWSFP = soursop fruit peel.

Given that activation of the polyol pathway consumes intracellular NADPH, the effect of soursop-enriched fecal metabolites on glutathione metabolism was subsequently evaluated. Fructose-induced adipotoxicity significantly (*p* < 0.05) disrupted glutathione homeostasis, as evidenced by reduced GPx, GR activities and intracellular GSH levels compared with the normal control, as shown in Figure 4A–C. Treatment with soursop-enriched fecal metabolites significantly (*p* < 0.05) restored all three parameters in a dose-dependent manner.

GPx activity was significantly (*p* < 0.05) suppressed following fructose exposure but was significantly elevated by SWSF, SWSFP and gallic acid (Figure 4A). SWSFP consistently exhibited the strongest stimulatory effect, restoring GPx activity above the normal control at the higher concentrations. GPx constitutes the first enzymatic defense against hydrogen peroxide and lipid hydroperoxides by utilizing GSH as an electron donor [70]. Therefore, restoration of GPx activity indicates enhanced detoxification of ROS and reduced susceptibility of adipocytes to oxidative damage. The superior response observed with SWSFP may reflect greater enrichment of metabolites capable of enhancing cellular antioxidant defenses through the gut–adipose axis.

Similarly, fructose significantly (*p* < 0.05) decreased GR activity, whereas all treatments restored the enzyme activity (Figure 4B). Although SWSF and SWSFP produced progressive improvements with increasing concentration, gallic acid exhibited the greatest restoration at higher concentrations. GR regenerates GSH from oxidized glutathione (GSSG) using NADPH, thereby maintaining the intracellular reducing environment. As excessive activation of the polyol pathway depletes NADPH through aldose reductase activity (Figure 3), the recovery of GR activity suggests preservation of NADPH availability and restoration of glutathione recycling [71]. Improved GR activity therefore complements the inhibition of the polyol pathway previously observed.

Consistent with these results, intracellular GSH levels were significantly depleted following fructose treatment but were significantly restored by all treatments (Figure 4C). SWSFP produced the highest increase in GSH level, followed by SWSF, whereas gallic acid exhibited only modest improvement. GSH is the principal non-enzymatic antioxidant in adipocytes and plays a central role in maintaining redox homeostasis, detoxifying lipid hydroperoxides and preserving insulin signaling [72]. Restoration of GSH therefore indicates improved antioxidant capacity and enhanced resistance to oxidative injury associated with fructose-induced adipotoxicity.

The recovery of GPx, GR and GSH demonstrates coordinated restoration of the glutathione redox cycle by soursop-enriched fecal metabolites. This result extends the observations in Figure 3, where suppression of the polyol pathway would be expected to reduce NADPH consumption, thereby facilitating glutathione regeneration. Within the context of the gut–adipose axis, soursop-enriched fecal metabolites appear to preserve intracellular redox balance by maintaining the glutathione system, thereby limiting oxidative stress and improving adipocyte metabolic function. Restoration of glutathione metabolism also establishes a favorable intracellular environment for subsequent detoxification of reactive dicarbonyls through the glyoxalase system, providing a mechanistic basis for the evaluation of GLO1 activity and carbonyl stress in the following section.

### 4.6. The Glyoxalase System

**Figure 5:**
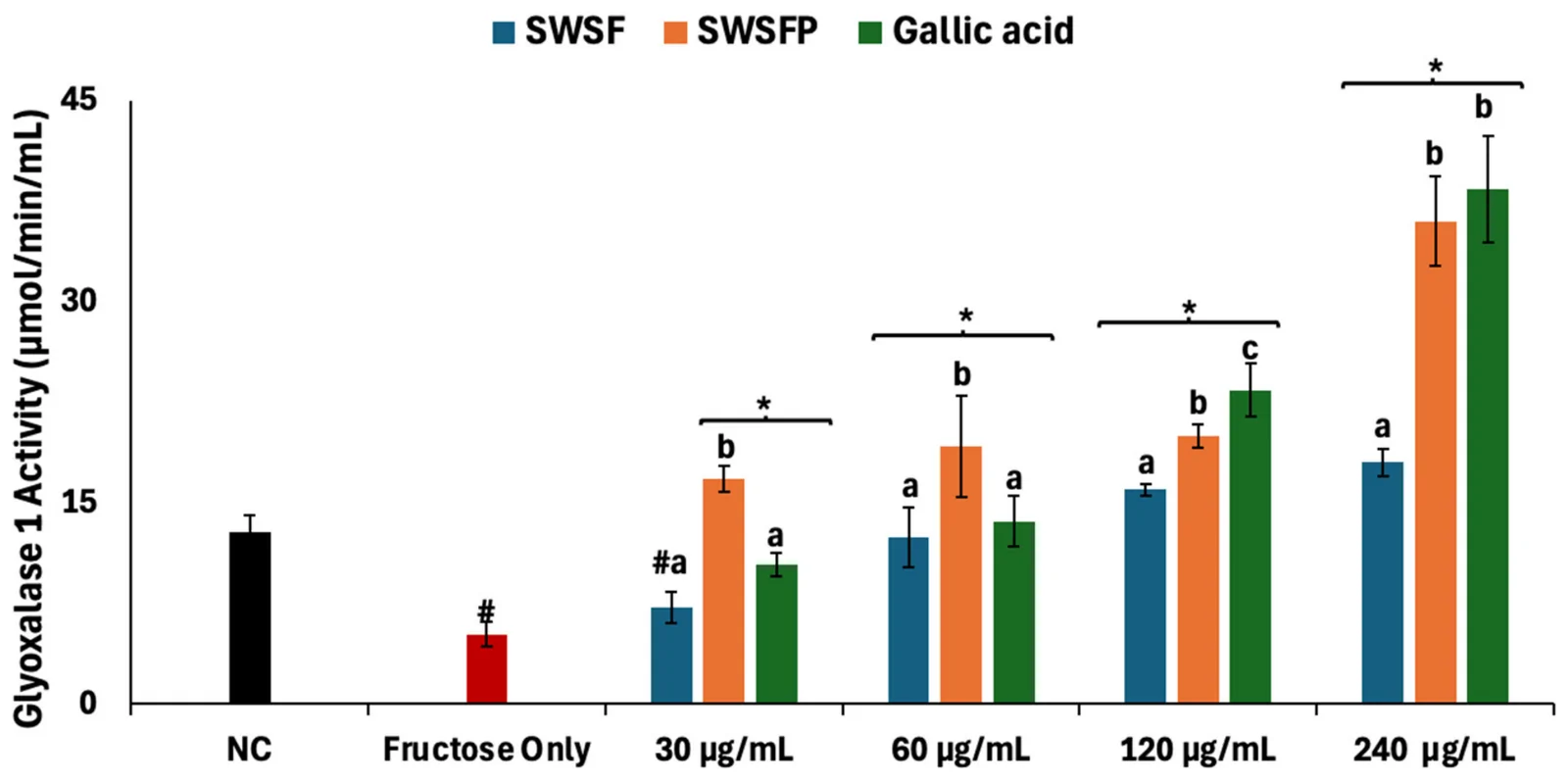
Effect of soursop-enriched fecal metabolites on GLO-1 activity in fructose-induced adipotoxicity. Data = mean ± SD; n = 3. * Statistically significant (*p* < 0.05) compared to fructose only; # statistically significant (*p* < 0.05) compared to NC. ^a,b,c^ Values with different letters above the bars are significantly (*p* < 0.05) different from each other. NC = normal control; SWSF = soursop fruit; SWSFP = soursop fruit peel.

To determine whether restoration of glutathione metabolism translated into improved detoxification of reactive carbonyl species, GLO-1 activity was evaluated following treatment with soursop-enriched fecal metabolites. Fructose-induced adipotoxicity significantly (*p* < 0.05) suppressed GLO-1 activity compared with the normal control (Figure 5), indicating impaired detoxification of reactive dicarbonyl compounds under conditions of metabolic stress. Treatment with SWSF, SWSFP and gallic acid significantly (*p* < 0.05) elevated GLO-1 activity in a dose-dependent manner, although the magnitude of recovery differed among the treatments.

At 30 μg/mL, SWSFP exhibited the highest increase in GLO-1 activity, followed by gallic acid and SWSF. Progressive increases in enzyme activity were observed as treatment concentrations increased. At 120 and 240 μg/mL, SWSFP and gallic acid exhibited greater stimulation of GLO-1 than SWSF, with enzyme activity exceeding that of the normal control at the highest concentration. These results indicate that soursop-enriched fecal metabolites significantly restored the glyoxalase defense system disrupted by fructose-induced adipotoxicity.

GLO-1 is the rate-limiting enzyme of the glyoxalase pathway and catalyzes the glutathione-dependent detoxification of methylglyoxal, a highly reactive dicarbonyl generated from fructose from the polyol pathway [73]. Suppression of GLO-1 promotes methylglyoxal accumulation, resulting in excessive formation of advanced glycation end-products (AGEs), carbonyl stress, mitochondrial dysfunction, and impaired insulin signaling [73,74]. In adipose tissue, these events accelerate lipid accumulation, chronic inflammation and adipocyte dysfunction, thereby contributing to the progression of insulin resistance and metabolic disease [75].

The restoration of GLO-1 activity is linked to the improvements in glutathione metabolism observed in Figure 4. GSH serves as an essential cofactor for GLO-1; thus, the recovery of intracellular GSH levels together with enhanced GR and GPx activities would be expected to facilitate efficient glyoxalase-mediated detoxification of methylglyoxal [76]. Furthermore, inhibition of the polyol pathway (Figure 3) indicates reduced NADPH depletion, thereby supporting continuous regeneration of GSH and sustaining GLO-1 activity. These coordinated responses indicate that soursop-enriched fecal metabolites restored multiple interconnected antioxidant systems rather than a single enzymatic pathway. Within the context of the gut–adipose axis, these results suggest that the enriched metabolites enhanced the intrinsic carbonyl detoxification capacity of adipocytes. Restoration of GLO-1 activity suggests that the enriched metabolites reduce methylglyoxal accumulation, limit AGE formation and attenuate carbonyl stress, thereby protecting adipocytes from glucotoxic and lipotoxic injury. Therefore, providing a critical mechanistic link between improved gut-derived metabolite composition and the preservation of adipocyte metabolic function.

### 4.7. Nucleotide Metabolism

**Figure 6:**
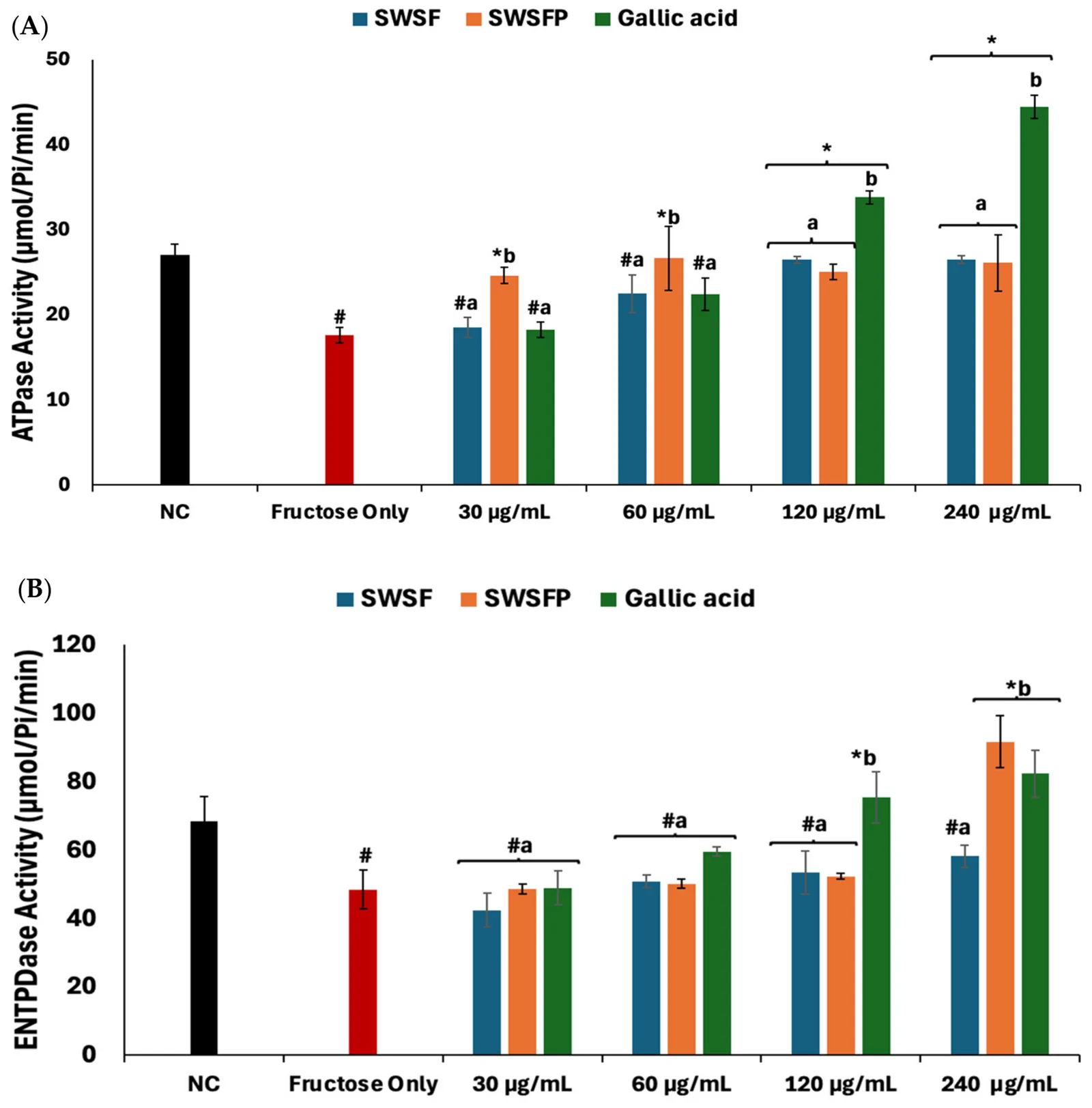
Effect of soursop-enriched fecal metabolites on (**A**) ATPase and (**B**) ENTPDase activities in fructose-induced adipotoxicity. Data = mean ± SD; n = 3. * Statistically significant (*p* < 0.05) compared to fructose only; # statistically significant (*p* < 0.05) compared to NC. ^a,b^ Values with different letters above the bars are significantly (*p* < 0.05) different from each other. NC = normal control; SWSF = soursop fruit; SWSFP = soursop fruit peel.

Given the restoration of glucose metabolism, glutathione homeostasis, and GLO-1 activity by soursop-enriched fecal metabolites, the effects of these metabolites on purinergic signaling were subsequently evaluated by determining ATPase and ENTPDase activities. As shown in Figure 6A,B, fructose-induced adipotoxicity significantly (*p* < 0.05) suppressed the activities of both enzymes compared with the normal control, indicating impaired ATP hydrolysis and dysregulated extracellular nucleotide metabolism. Treatment with SWSF, SWSFP, and gallic acid significantly (*p* < 0.05) restored ATPase and ENTPDase activities in a dose-dependent manner.

ATPases regulate ATP hydrolysis, thereby maintaining intracellular energy homeostasis and limiting excessive ATP accumulation [77]. Reduced ATPase activity under fructose-induced metabolic stress may impair ATP turnover, promote mitochondrial dysfunction, and increase extracellular ATP release. Extracellular ATP functions as a danger-associated molecular pattern (DAMP), activating purinergic receptors and initiating inflammatory signaling that contributes to adipocyte dysfunction and insulin resistance [78]. Restoration of ATPase activity therefore suggests improved cellular energy metabolism and attenuation of ATP-mediated inflammatory responses.

ENTPDases hydrolyze extracellular ATP and ADP to AMP, thereby regulating purinergic signaling and preventing sustained activation of ATP-sensitive inflammatory pathways [79]. SWSFP demonstrated the greatest restoration at the highest concentration, while gallic acid also produced marked improvements at 120 and 240 μg/mL (Figure 6B). Enhanced ENTPDase activity may indicate accelerated clearance of extracellular ATP, reducing activation of pro-inflammatory purinergic receptors and limiting chronic inflammatory signaling within adipose tissue.

These results complement the preceding observations on glucose metabolism (Figure 2), polyol pathway inhibition (Figure 3), glutathione metabolism (Figure 4) and GLO-1 activity (Figure 5). Restoration of intracellular glucose handling reduces metabolic overload, while preservation of glutathione metabolism and glyoxalase activity suppress oxidative and carbonyl stress. Improved purinergic enzyme activities further extend these protective effects by maintaining ATP homeostasis and suppressing ATP-driven inflammatory signaling [77,79]. These coordinated responses indicate that soursop-enriched fecal metabolites concomitantly restore multiple interconnected metabolic networks that are disrupted during fructose-induced adipotoxicity. Within the context of the gut–adipose axis, the enriched metabolites may regulate adipocyte function beyond glucose metabolism by maintaining extracellular nucleotide homeostasis. Improved ATPase and ENTPDase activities suggest reduced ATP-mediated inflammation, improved mitochondrial energy metabolism and enhanced insulin responsiveness [77,79], thereby suppressing the metabolic and inflammatory disturbances associated with adipocyte dysfunction.

### 4.8. Inflammatory Lipid Metabolism

**Figure 7:**
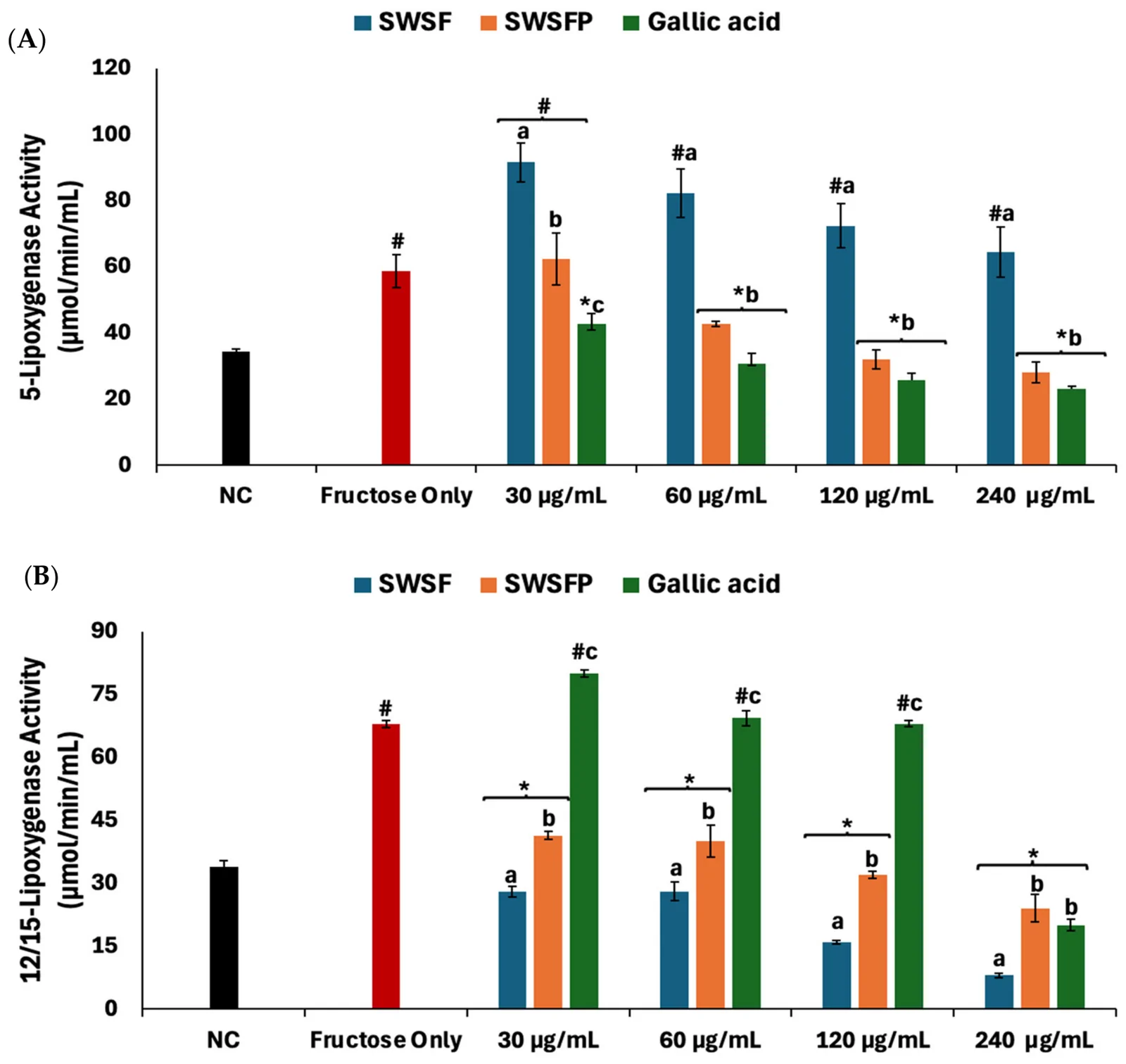
Effect of soursop-enriched fecal metabolites on (**A**) 5-lipoxygenase and (**B**) 12/15-lipoxygenase activities in fructose-induced adipotoxicity. Data = mean ± SD; n = 3. * Statistically significant (*p* < 0.05) compared to fructose only; # statistically significant (*p* < 0.05) compared to NC. ^a,b,c^ Values with different letters above the bars are significantly (*p* < 0.05) different from each other. NC = normal control; SWSF = soursop fruit; SWSFP = soursop fruit peel.

To determine whether the restoration of purinergic signaling led to attenuation of inflammatory lipid metabolism, the activities of 5-LOX and 12/15-LOX were evaluated following treatment with soursop-enriched fecal metabolites. Fructose-induced adipotoxicity significantly (*p* < 0.05) increased the activities of both enzymes compared with the normal control, as shown in Figure 7A,B, indicating enhanced metabolism of polyunsaturated fatty acids towards pro-inflammatory lipid mediators. Treatment with SWSF, SWSFP and gallic acid significantly suppressed the activities of both enzymes in a dose-dependent manner.

Fructose significantly (*p* < 0.05) elevated 5-LOX activity, whereas treatment with soursop-enriched fecal metabolites significantly suppressed the enzyme activity (Figure 7A). SWSF consistently exhibited the greatest inhibitory activity across all concentrations, restoring enzyme activity to near-normal at 240 μg/mL. SWSFP also significantly (*p* < 0.05) suppressed 5-LOX activity, albeit to a lesser extent, whereas gallic acid showed the least inhibition. 5-LOX catalyzes the conversion of arachidonic acid into leukotrienes and other bioactive lipid mediators that promote inflammatory cell recruitment, oxidative stress, and insulin resistance [80]. Persistent activation of 5-LOX in adipose tissue contributes to chronic low-grade inflammation, adipocyte hypertrophy and impaired insulin signaling [81], all of which are hallmarks of fructose-induced adipotoxicity. Therefore, inhibition of 5-LOX suggests attenuation of inflammatory lipid signaling and improved adipocyte metabolic function.

A similar trend was observed for 12/15-LOX activity (Figure 7B). Fructose significantly (*p* < 0.05) elevated enzyme activity, whereas all treatments reduced its activity, with SWSF exhibiting the greatest inhibition at higher concentrations. Gallic acid exhibited relatively limited suppression, indicating that the fermentation-derived metabolites exerted greater anti-inflammatory effects than the reference compound. The 12/15-LOX pathway generates hydroxyeicosatetraenoic acids (HETEs) and oxidized lipid mediators that stimulate oxidative stress, macrophage infiltration and inflammatory gene expression in adipose tissue [82]. Enhanced 12/15-LOX activity has also been linked to impaired insulin signaling and progressive adipocyte dysfunction [83]. Thus, suppression of this pathway suggests reduced lipid peroxidation and preservation of adipocyte metabolic integrity.

The inhibition of both lipoxygenase isoforms complements the metabolic improvements observed in the preceding figures. Restoration of glucose metabolism (Figure 2), suppression of the polyol pathway (Figure 3), preservation of glutathione homeostasis (Figure 4), enhancement of GLO-1 activity (Figure 5) and restoration of purinergic enzyme activities (Figure 6) collectively suppress the intracellular metabolic stress that drives inflammatory lipid signaling. Furthermore, the metabolomic analysis (Figure 1) demonstrated enrichment of pathways associated with arachidonic acid metabolism, α-linolenic and linoleic acid metabolism, and fatty acid metabolism, indicating that fecal-enriched metabolites can remodel polyunsaturated fatty acid metabolism. These alterations may have contributed to the reduced availability of pro-inflammatory lipid substrates and the suppression of lipoxygenase activities observed in the present study. Within the framework of the gut–adipose axis, these results suggest that the enriched metabolites regulate adipocyte inflammation by modulating lipid mediator biosynthesis. By suppressing both 5-LOX and 12/15-LOX, the soursop-enriched fecal metabolites may reduce the production of leukotriene and HETE, thereby limiting inflammatory signaling, oxidative stress and insulin resistance. This coordinated modulation of glucose metabolism, redox homeostasis, purinergic signaling and lipoxygenase activity suggests strong mechanistic evidence that remodeling of gut-derived metabolites contributes to the attenuation of fructose-induced adipotoxicity.

### 4.9. Adipose Tissue Metabolomics

**Figure 8:**
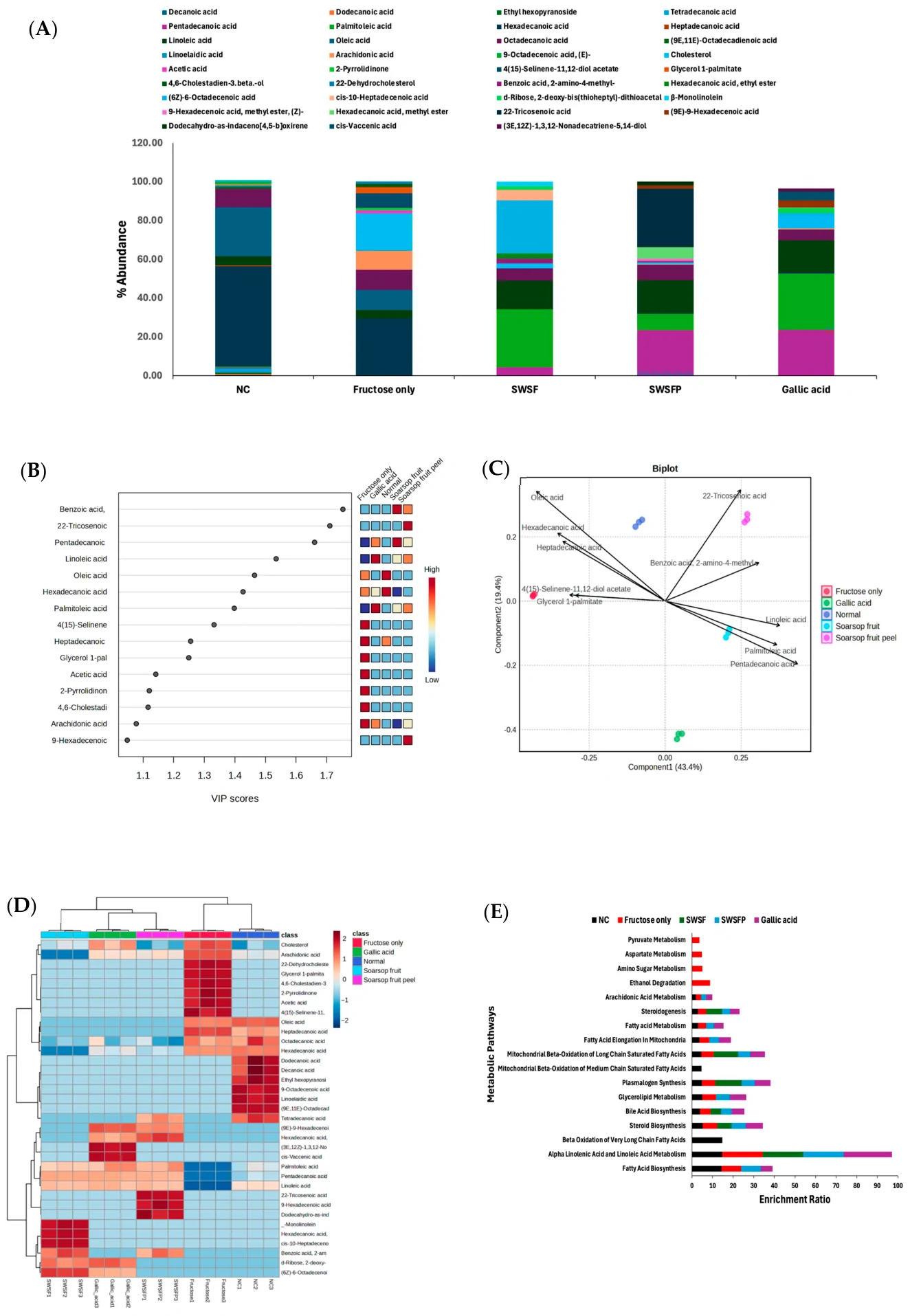
(**A**) Effect of soursop-enriched fecal metabolites on adipose metabolites in fructose-induced adipotoxicity; (**B**) VIP scores; (**C**) PLS biplot; (**D**) heatmap; and (**E**) pathway enrichment of adipose metabolites. NC = normal control; SWSF = soursop fruit; SWSFP = soursop fruit peel.

To further elucidate the mechanisms underlying the protective effects of soursop-enriched fecal metabolites against fructose-induced adipotoxicity, untargeted GC–MS metabolomic analysis was performed on adipose tissue following treatment (Figure 8A–E). Fructose significantly altered the adipose metabolite profile compared with the normal control, whereas treatment with SWSF, SWSFP and gallic acid substantially remodeled the adipose metabolome, indicating that soursop-enriched fecal metabolites exerted downstream metabolic effects within adipose tissue.

The relative abundance profiles (Figure 8A) demonstrated marked alterations in fatty acids, sterols and lipid-derived metabolites among the experimental groups. Fructose exposure was characterized by increased abundance of palmitic acid, oleic acid, hexadecanoic acid, glycerol-1-palmitate and methylated fatty acid derivatives, reflecting enhanced lipid accumulation and disrupted fatty acid metabolism. Treatment with SWSF and SWSFP shifted the metabolite profile towards increased proportions of linoleic acid, arachidonic acid, cholesterol, cis-vaccenic acid and other unsaturated lipid metabolites, suggesting restoration of lipid remodeling and membrane homeostasis. These alterations indicate that the enriched metabolites substantially modified adipocyte lipid composition through the gut–adipose metabolic axis.

Variable importance in projection (VIP) analysis identified benzoic acid, 22-tricosenoic acid, pentadecanoic acid, linoleic acid, oleic acid, hexadecanoic acid, palmitoleic acid, heptadecanoic acid, glycerol-1-palmitate and acetic acid as the principal metabolites contributing to group discrimination (Figure 8B). The PLS biplot (Figure 8C) further demonstrated distinct clustering of the treatment groups, with fructose-treated adipocytes clearly separated from the normal and treated groups. SWSF and SWSFP occupied distinct metabolic spaces, indicating that the two fermentation products differentially remodeled adipose metabolism. Heatmap analysis (Figure 8D) also revealed treatment-specific metabolite signatures, confirming that soursop-derived fermentation metabolites reversed most of the fructose-induced metabolic disturbances.

The identified metabolites have important implications for glucose and lipid homeostasis. Linoleic acid and arachidonic acid are central intermediates in polyunsaturated fatty acid metabolism, regulating membrane fluidity, insulin responsiveness, and inflammatory lipid signaling [84]. Pentadecanoic acid has emerged as a biomarker of improved metabolic health and has been associated with enhanced insulin sensitivity and reduced risk of metabolic syndrome [85]. Excessive accumulation of palmitic acid, hexadecanoic acid, and glycerol-1-palmitate contributes to lipotoxicity, endoplasmic reticulum stress, and impaired insulin signaling [86]. Likewise, oleic acid and cis-vaccenic acid participate in triglyceride turnover and membrane lipid remodeling [87,88], while cholesterol is essential for membrane integrity and insulin receptor organization [89]. These observed alterations suggest a shift from a lipotoxic metabolic phenotype towards improved lipid homeostasis following treatment with soursop-enriched fecal metabolites.

Pathway enrichment analysis (Figure 8E) demonstrated that the altered adipose metabolites were predominantly associated with fatty acid biosynthesis, α-linolenic and linoleic acid metabolism, arachidonic acid metabolism, fatty acid metabolism, glycerolipid metabolism, fatty acid elongation in mitochondria, β-oxidation of long-chain saturated fatty acids, steroid biosynthesis and steroidogenesis. These pathways are fundamental regulators of fatty acid synthesis, lipid oxidation, membrane phospholipid remodeling and insulin sensitivity [90]. The enrichment of mitochondrial β-oxidation pathways suggests enhanced utilization of accumulated fatty acids, whereas modulation of glycerolipid and sterol metabolism indicates improved lipid turnover and membrane function [90]. Furthermore, remodeling of arachidonic acid metabolism is consistent with the reduced 5-LOX and 12/15-LOX activities observed in Figure 7, supporting attenuation of inflammatory lipid mediator synthesis.

These results may suggest compelling evidence that soursop-enriched fecal metabolites remodel adipose tissue metabolism via the gut–adipose axis. These metabolomic alterations complemented the restored glucose metabolism (Figure 2), suppressed the polyol pathway (Figure 3), improved glutathione metabolism (Figure 4), enhanced GLO-1 activity (Figure 5), normalized purinergic signaling (Figure 6), and attenuated lipoxygenase-mediated inflammation (Figure 7). Synergistically, these coordinated metabolic adaptations indicate that soursop-enriched fecal metabolites modulate adipocyte metabolism toward improved glucose utilization, enhanced lipid turnover, reduced inflammatory lipid signaling, and preservation of metabolic homeostasis, thereby mitigating fructose-induced adipotoxicity.

### 4.10. Proposed Mechanism

**Figure 9:**
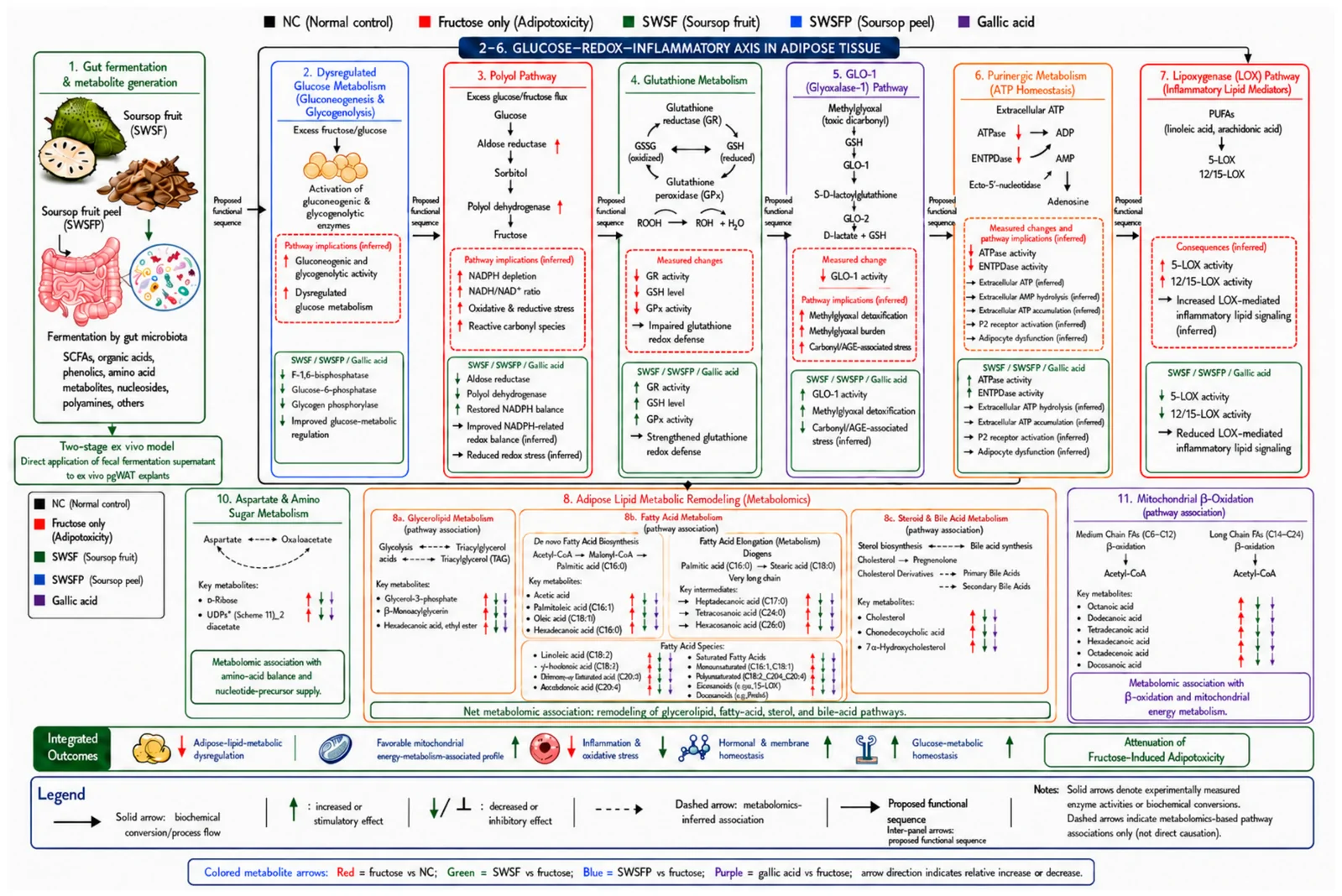
Schematic integrated pathway.

Based on the integration of biochemical assays and untargeted metabolomic pathway enrichment analyses performed in the present study, a mechanism was proposed (Figure 9). Fermentation of SWSF and SWSFP by the gut microbiota generates bioactive metabolites that modulate adipocyte metabolism after their transfer via the gut–adipose axis. These metabolites suppress aberrant activation of gluconeogenic and glycogenolytic enzymes, inhibit the polyol pathway, maintain glutathione metabolism and glyoxalase-1-mediated carbonyl detoxification, restore purinergic enzyme activities, and attenuate lipoxygenase-mediated inflammatory lipid signaling. These biochemical adaptations modulate adipose lipid metabolism by regulating glycerolipid metabolism, fatty acid biosynthesis and β-oxidation, arachidonic acid metabolism, plasmalogen synthesis, steroid and bile acid metabolism, and mitochondrial energy metabolism. The coordinated regulation of these interconnected pathways reduces oxidative stress, carbonyl stress, inflammation, lipid accumulation and insulin resistance, ultimately restoring glucose and lipid homeostasis and protecting against fructose-induced adipotoxicity.

## 5. Conclusions

This study demonstrates that gut microbial fermentation of soursop pulp and peel produces metabolites capable of protecting adipose tissue against fructose-induced adipotoxicity. Soursop-enriched fecal metabolites restored metabolic homeostasis by suppressing aberrant glucose metabolism, inhibiting the polyol pathway, preserving glutathione-dependent antioxidant defenses, enhancing glyoxalase-1 activity for carbonyl detoxification, normalizing purinergic enzyme activities, and attenuating lipoxygenase-mediated inflammatory lipid signaling. These biochemical improvements were accompanied by extensive remodeling of adipose and fecal metabolomes, particularly pathways involved in fatty acid biosynthesis, β-oxidation, glycerolipid metabolism, sterol metabolism, arachidonic acid metabolism, and mitochondrial energy metabolism. Taken together, these results indicate that modulation of the soursop–gut–adipose axis improves glucose and lipid homeostasis, suppresses oxidative, carbonyl, and inflammatory stress, and preserves adipocyte metabolic function in fructose-induced adipotoxicity. Although pooling the fecal samples provided a standardized fermentation inoculum, it precluded assessment of interindividual variation, while the untargeted GC–MS metabolite annotations require confirmation through targeted analysis using standards. Furthermore, the pgWAT ex vivo model does not fully reproduce intestinal absorption, systemic transport, hepatic metabolism, or endocrine and immune interactions. Nevertheless, the study provides mechanistic insights supporting further investigation of soursop as a potential functional food for metabolic disorders associated with adipose dysfunction. Subsequent in vivo studies, followed by appropriate clinical investigations, are warranted to validate these findings and identify the specific gut-derived metabolites responsible for the observed metabolic benefits.

## Figures and Tables

**Table 1:** GC-MS-identified compounds in soursop fruit and peel.

Compounds	SWSF (%)	SWSFP (%)
Acetic acid	24	ND
3,5-Dihydroxy-6-methyl-2,3-dihydro-4H-pyran-4-one	35	24
Decahydroquinoline-10-ol	20	ND
3,7-Dimethylimidazo [1,2-a]pyrimidine-2,5(1H,3H)-dione	10	10
6-methylpteridine-2,4,7-trione	3	ND
L-(+)-Ascorbic acid 2,6-dihexadecanoate	4	3.40
6-Octadecenoic acid, (Z)-	4	4.09
Decahydroquinolin-10-ol	ND	30.36
Octahydro-2(1H)-quinolinone	ND	21.38
Octadecanoic acid	ND	1.45
Glycerol 1-palmitate	ND	1.01
Glyceryl Monooleate	ND	1.40
13-Docosenamide, (Z)-	ND	1.64
Gamma-Sitosterol	ND	0.93

ND = not detected; SWSF = soursop fruit; SWSFP = soursop fruit peel.

## Data Availability

The original contributions presented in the study are included in the article; further inquiries can be directed to the corresponding authors.
